# Biochemical evidence for diverse strategies in the inner kinetochore

**DOI:** 10.1098/rsob.200284

**Published:** 2020-11-18

**Authors:** G. E. Hamilton, T. N. Davis

**Affiliations:** Department of Biochemistry, University of Washington, Seattle, WA, USA

**Keywords:** mitosis, kinetochore, centromere, genome, CENP-A

## Abstract

The kinetochore is a complex structure whose function is absolutely essential. Unlike the centromere, the kinetochore at first appeared remarkably well conserved from yeast to humans, especially the microtubule-binding outer kinetochore. However, recent efforts towards biochemical reconstitution of diverse kinetochores challenge the notion of a similarly conserved architecture for the constitutively centromere-associated network of the inner kinetochore. This review briefly summarizes the evidence from comparative genomics for interspecific variability in inner kinetochore composition and focuses on novel biochemical evidence indicating that even homologous inner kinetochore protein complexes are put to different uses in different organisms.

## 1. Introduction

Centromeres are one of the last great enigmas of the genome. Although specialized sites of microtubule attachment on chromosomes were first described by Flemming over a century ago [1], the linear assemblies of most human centromeres remain elusive to this day, nearly two decades after the official completion of the Human Genome Project [2–5]. Curiously, despite the fact that all centromeres share a common function—recruitment of kinetochore proteins, which mediate chromosome-microtubule attachments essential for cell division—they differ radically in size and sequence.

Some organisms, including the model organism *Caenorhabditis elegans,* have holocentric chromosomes, which bind microtubules along their entire lengths through widespread kinetochore activity [6–8]. But in most organisms, kinetochore activity is restricted to a single, relatively small region of each chromosome, termed a regional centromere [9]. The simplest centromeric sequences are found in *Saccharomyces cerevisiae* and related budding yeast; these centromeres are defined by a minimal sequence of only about 125 base pairs containing three conserved centromere-determining elements (CDEI, CDEII and CDEIII) [10–12]. Such ‘point' centromeres are exceptional for both their diminutive size and sequence conservation [13,14]. In general, centromeric DNA is highly repetitive and AT-rich, though the size and sequence of regional centromeres vary tremendously between species; regional centromeres can be as small as a few kilobases, or they can span megabases [15,16]. Centromere sequence and location can even vary at the population level within species [4,17–19]. There is tremendous variation in the DNA sequences on which kinetochores assemble.

However, it should be noted that this traditional definition of the centromere excludes the more recently recognized contributions of ‘pericentric' chromatin, which forms specialized, conserved structures with important biophysical properties (reviewed in [20]). Lawrimore & Bloom [20] postulate that highly looped, ‘bottlebrush' structures may be formed either by a single regional centromere [21] or a clustered ensemble of point centromeres and their pericentromeric chromatin [22,23]. Although there are different models of the exact structure of pericentromeric chromatin [24,25], there is a growing consensus that such structures are significant and may represent a conserved centromeric architecture that belies the genetic variety of centromere sequence [20,24].

Furthermore, genetic sequence alone does not a centromere make. Both genetic and epigenetic factors contribute to centromere identity, and their relative importance varies between organisms [16]. Budding yeast rely heavily on genetic determinants of centromere identity [11,16,26]. But in the vast majority of organisms studied thus far,^1^ centromeres are primarily specified in an epigenetic manner by the presence of a centromere-specific histone H3 variant termed CENP-A [31,32] (table 1). CENP-A positioning is epigenetically propagated, because existing CENP-A nucleosomes template new CENP-A deposition by recruiting necessary factors such as HJURP, CENP-C and the Mis18 complex in vertebrates [33–39]. Indeed, in higher eukaryotes, centromere inheritance appears minimally affected by DNA sequence; centromeres can migrate into neighbouring regions, a phenomenon known as ‘centromere drift' [16,40]. Experimentally mistargeted CENP-A can incorporate into non-centromeric chromatin (including both heterochromatin and actively transcribed euchromatin) and recruit kinetochore proteins, creating ‘neocentromeres' that can be transmitted mitotically [33,41,42]. Neocentromeres also form on non-repetitive DNA without experimental manipulation—over 100 different neocentromeres have been identified in living humans [43,44]. In several such cases, the original centromeres were permanently silenced without rearrangement or deletion of formerly centromeric repeat DNA sequences, by poorly understood epigenetic mechanisms [45,46]. In other words, in humans, the presence CENP-A at a given chromosomal locus, unlike the underlying DNA sequence, is both necessary and sufficient for mitotic centromere function.

**Table 1.** Distribution and importance (if known) of inner kinetochore proteins in organisms with relatively well-characterized kinetochores. Grey shading indicates that no homologue has yet been identified in this organism. Blue shading indicates that a homologue has been identified, but not yet been shown to be essential. Green shading indicates that a homologue is both present and essential for viability. Orange shading indicates the primary path of outer kinetochore recruitment. Yellow shading indicates a secondary path(s) of outer kinetochore recruitment. Future biochemical characterization of diverse inner kinetochores will necessitate updating this table with novel findings about each protein's homologues, essentiality and function.

	fungus	fungus	fungus	nematode	insect	mammal	bird	insect	amphibian	mammal	plant
CENP-	*S. cerevisiase*	*S. pombe*	*M. circinelloides*	*C. elegans*	*B. mori*	*H. sapiens*	*G. gallus*	*D. melanogaster*	*X. laevis/tropicalis*	*M. musculus*	*A. thaliana*
**A**	Cse4	Cnp1		HCP-3		CENP-A	CenpA	CID	CENP-A	Cenpa	cenH3/HTR12
**C**	Mif2	Cnp3		HCP-4		CENP-C	CENPC	CENP-C	CENP-C	Cenpc1	atCENP-C
**H**	Mcm16	Fta3	CENP-H		GSSPFG00011797001?	CENP-H	CENPH		CENP-H	Cenph	
**I**	Ctf3	Mis6	CENP-I		KWMTBOMO02221	CENP-I	CENPI		CENP-I	Cenpi	
**K**	Mcm22	Sim4	CENP-K		LOC101741561	CENP-K	CENPK		CENP-K	Cenpk	
**L**	Iml3	Fta1	CENP-L		KMWTBOMO11447	CENP-L	CENPL		CENP-L	Cenpl	
**M**			CENP-M		LOC101745870	CENP-M	CENPM		CENP-M	Cenpm	
**N**	Chl4	Mis15	CENP-N		KWMTBOMO06206	CENP-N	CENPN		CENP-N	Cenpn	
**O**	Mcm21	Mal2	CENP-O		KWMTBOMO14835?	CENP-O	CENPO		CENP-O	Cenpo	NP_568235.1
**P**	Ctf19	Fta2	CENP-P		KWMTBOMO09290?	CENP-P	CENPP		CENP-P	Cenpp	
**Q**	Okp1	Fta7				CENP-Q	CENPQ			Cenpq	
**U**	Ame1	Mis17				CENP-U	CENPU		CENP-U	Cenpu	
**R**						CENP-R			CENP-R	Cenpr	
**S**	Mhf1	Mhf1	CENP-S	Y48E1C.1		CENP-S	CENPS		CENP-S	Cenps	NP_199906.1
**T**	Cnn1	Cnp20	CENP-T		CENP-T	CENP-T	CENPT		CENP-T	Cenpt	
**W**	Wip1	Wip1	CENP-W			CENP-W	CENPW		CENP-W	Cenpw	
**X**	Mhf2	Mhf2	CENP-X	F35H10.5		CENP-X	CENPX		CENP-X		NP_001323102
**—**	Nkp1	Fta4									
**—**	Nkp2	Cnl2									

Despite a shared method of epigenetic centromere specification and potential structural commonalities in the pericentromere, the sheer diversity of centromere size and sequence indicates that these loci are rapidly evolving [47,48].

The paradox of absolutely essential, yet poorly conserved centromeres defied explanation until it was placed in the intellectual framework of meiotic drive [49]. This novel view of centromeric chromatin as selfish genetic elements, competing with their homologues for inclusion in gametes during asymmetrical meiotic divisions, explained the rapid evolution of centromeric DNA, and the influential theory became known as ‘centromere drive' [49–51].

The kinetochore, too, is a complex structure whose function is absolutely essential. Unlike the centromere, the kinetochore at first appeared remarkably well conserved from yeast to humans, especially the microtubule-binding outer kinetochore. However, recent efforts towards biochemical reconstitution of diverse kinetochores challenge the notion of a similarly conserved architecture for the constitutively centromere-associated network (CCAN) of the inner kinetochore. This review briefly summarizes the evidence from comparative genomics for interspecific variability in inner kinetochore composition and focuses on novel biochemical evidence indicating that even homologous inner kinetochore protein complexes are put to different uses in different organisms. We confine our discussion to the mitotic functions of inner kinetochore proteins, because their contributions to meiosis are much less well understood [52–54].

## 2. Genetic evidence

Two types of genetic evidence suggest an evolutionarily labile inner kinetochore: evidence that CCAN proteins are rapidly evolving on the sequence level and evidence that different genomes contain different subsets of kinetochore proteins.

There is ample evidence that many kinetochore proteins are rapidly evolving. Comparing the rate of nonsynonymous substitutions (dN) to synonymous ones (dS) is a widely used metric for quantification of selection pressure on a given gene. Kinetochore protein genes have an average dN/dS value four times greater than that of anaphase-promoting complex proteins [12,30]. Although ascomycete kinetochore proteins are largely conserved with those of metazoans (hence the tremendous value of *S. cerevisiae* as a model organism in the field of kinetochore biology), even within the fungal kingdom inner kinetochore proteins are poorly conserved at the primary sequence level [55]. CENP-A, the foundation of the kinetochore, is also rapidly evolving [51,56,57], whereas conventional histones are among the best-conserved proteins in eukaryotic genomes.

Comparative genomics confirm that, throughout eukaryotic evolution, many inner kinetochore proteins have been lost as a group numerous times [30]. But major components of the outer kinetochore, including the Ndc80 complex (Ndc80c), Mis12c^MIND^ and the Spc105 complex (Spc105c), have been largely conserved throughout eukaryotic evolution [12,30,58].

Ndc80c, the primary microtubule-binding element of the kinetochore, is the most striking example of outer kinetochore conservation. It is present in nearly every eukaryotic genome surveyed [30], with the possible exception of kinetoplastids [28,58].

An important caveat to all of these genomic studies is that while the ‘hits' can be informative, the ‘misses' must be viewed sceptically. Failure to detect a homologue could indicate that it is indeed absent from the queried genome, but it could also indicate that a divergent primary sequence has allowed the homologue to escape detection. Much more persuasive are biochemically validated similarities and differences between kinetochores from different lineages.

## 3. Biochemical evidence

Biochemical and biophysical characterization of the kinetochore began with its microtubule-binding elements, where remarkable conservation was observed. All kinetochores that have been robustly characterized thus far couple to microtubules through a combination of Ndc80c and either Dam1c or the Ska complex [59–64], functional analogues with inversely correlated phylogenetic distributions [65]. Together, Ndc80c and either Dam1c or the Ska complex perform the most fundamental function of the kinetochore: harnessing the force of depolymerizing microtubules [60,64,66–70]. To date, every well-characterized kinetochore relies on a homologue of Ndc80c for microtubule binding (with the possible exception of kinetoplastids [28,71]).

**Figure 1.**
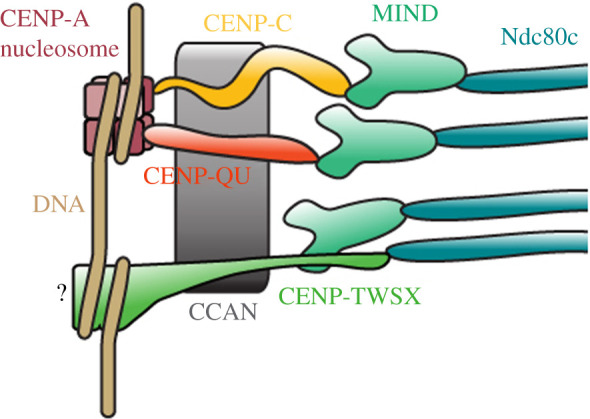
Three strategies of Ndc80c recruitment have been described biochemically in different organisms. Some organisms, notably ascomycetes, recruit Ndc80c primarily through CENP-QU homologues. In others, such as *D. melanogaster*, the CENP-C-based recruitment pathway dominates. Another set of organisms, including *G. gallus*, relies most heavily on CENP-TW for Ndc80c recruitment. CENP-QU and CENP-C bind centromeric nucleosomes directly and recruit Ndc80c through Mis12c^MIND^. CENP-TW, on the other hand, can bind Ndc80c both directly and through Mis12c^MIND^. CENP-TW is recruited to the kinetochore by upstream components of the CCAN (although it has been posited that CENP-TWSX could bind DNA directly in some organisms).

But there are several different strategies by which the inner kinetochore may recruit Ndc80c and transmit force to the centromere. Thus far, three types of inner kinetochore have been described biochemically (figure 1). There are CENP-C-dependent inner kinetochores (e.g. *Drosophila melanogaster*), CENP-QU-dependent inner kinetochores (e.g. *S. cerevisiae*) and CENP-T-dependent inner kinetochores. This last category can be subdivided into those that contain CENP-A homologues (e.g. *Gallus gallus*) and those that do not (e.g. *Bombyx mori*). The authors do not mean to suggest that these three categories encompass all the extant diversity of inner kinetochore architecture, merely those that have been described biochemically to date. For instance, the divergent kinetochores of kinetoplastids likely represent still a fourth type of inner kinetochore, but a biochemical dissection of that system is still ongoing.

### 3.1. CENP-C-based inner kinetochores

Identifying the most critical component of the highly simplified *D. melanogaster* inner kinetochore was relatively easy—CENP-C is the only candidate. The *D. melanogaster* genome lacks homologues to all other inner kinetochore proteins except CENP-A [30,72–74]. Although several insect lineages have lost their CENP-A homologues, *D. melanogaster* is not one of them [27].

Kinetochore formation in *Drosophila* cells depends absolutely on the presence of CENP-C [75]. It has also been experimentally validated both *in vivo* and *in vitro*, that CENP-C bridges CENP-A and Mis12c^MIND^ [76–78]. Targeting the N-terminus of CENP-C to centromeres is sufficient to recruit Mis12c^MIND^, Ndc80c and other outer kinetochore components to these ectopic loci [77]. Using both FRET- and talin-vinculin-based force sensors, it has been demonstrated that force is transmitted through CENP-C in mitotic *D. melanogaster* cells [79].

*Drosophila melanogaster* is not the only organism to have lost most of the CCAN. Examining the evolutionary dynamics of the kinetochore, van Hooff *et al*. [30] found that most of the CCAN evolved as an evolutionary unit that has been lost from many lineages. The exceptions were CENP-R, a recent invention in animals, CENP-S and -X, which have non-kinetochore functions in DNA repair [80,81] and CENP-C. Because CENP-C is the only widely retained CCAN component without a non-kinetochore function, the simplified, CENP-C-based CCAN may be a widespread inner kinetochore architecture. Kinetochore assembly on the holocentric chromosomes of *C. elegans* relies absolutely upon the CENP-C homologue HCP-6; its depletion phenocopies depletion of CENP-A [82,83].

### 3.2. CENP-QU-based inner kinetochores

A pioneering model organism in the field of kinetochore biology, *S. cerevisiae* is also an interesting case in terms of its relationship to the theory of centromere drive. There is conflicting evidence as to whether or not budding yeast undergo asymmetrical meiotic or postmeiotic divisions [84,85], so it is unclear if budding yeast centromeres and inner kinetochores have been shaped by this type of meiotic intragenomic conflict. Centromere sequences in closely related yeast species are rapidly evolving [86–88], but it is unknown if this is the result of centromere drive.

Budding yeast have 16 inner kinetochore proteins, of which only three are essential [89]. These essential proteins are CENP-Q^Okp1^ and CENP-U^Ame1^ [90], which form a dimeric subcomplex, and CENP-C^Mif2^ [91–93]. CENP-QU^Okp1/Ame1^ and CENP-C^Mif2^ bind both DNA and Mis12c^MIND^ [94], although they cannot bind to the same Mis12c^MIND^ simultaneously [95]. It has recently been demonstrated that CENP-QU^Okp1/Ame1^ and CENP-C^Mif2^ are independently capable of transmitting mitotically relevant forces between Mis12c^MIND^ and a centromeric nucleosome [96], but while the Mis12c^MIND^-binding residues of CENP-U^Ame1^ are essential, the Mis12c^MIND^-binding N-terminus of CENP-C^Mif2^ is not [94]. The association of CENP-QU^Okp1/Ame1^ with centromeric nucleosomes is regulated by post-translational modifications to the N-terminus of CENP-A^Cse4^ [97]. The CENP-QU-based inner kinetochore appears to be conserved among ascomycetes, as both CENP-QU homologues Fta7 and Mis17 are essential in *Schizosaccharomyces pombe*, which is quite phylogenetically distant from *S. cerevisiae* [98,99].

The role of CENP-QU in other organisms is less clear. Human CENP-Q was reported to bind microtubules, but not Mis12c^MIND^ [100,101]. Although CENP-U is essential during mammalian embryogenesis [102], its essential function could be something other than Mis12c^MIND^ recruitment. For example, CENP-U is reported to recruit the mitotic kinase Plk1 to kinetochores [103,104], and CENP-Q is required for loading the kinesin CENP-E onto kinetochores [105].

The diverse roles of CENP-QU in different organisms reflect this subcomplex's unique evolutionary history. In many lineages, the inner kinetochore has been simplified by the loss of genes over evolutionary time [30,73]. CENP-QU is the rare exception to that rule: an inner kinetochore subcomplex essential in some organisms, yet inferred to have been absent from the last eukaryotic common ancestor [30]. CENP-QU (and inessential inner kinetochore components Nkp1 and Nkp2) likely arose through the duplication of an ancestral Mis12^MIND^ complex [106]. Thus CENP-QU is a relatively recent addition to the inner kinetochore. Although it is essential in organisms like *S. cerevisiae*, CENP-QU has been lost recurrently throughout the fungal kingdom [29].

In budding yeast, CENP-T is an inessential protein [107–110] whose deletion causes only a mild defect in chromosome transmission fidelity [111]. Kinetochore localization of CENP-T depends on nearly every other inner kinetochore component [112,113]. Intriguingly, CENP-T becomes essential when interactions between Mis12c^MIND^ and the inner kinetochore are disrupted [112], suggesting that it might serve as a backup pathway for recruitment of the outer kinetochore in budding yeast. This stands in sharp contrast to the essential role of CENP-T in several other organisms.

### 3.3. CENP-T-based inner kinetochores

The third class of inner kinetochores that has been described to date is the CENP-T-based inner kinetochore. Whereas CENP-QU and CENP-C interact with both CENP-A and DNA [94], CENP-T interacts directly with the latter, but not the former [114]. CENP-T and CENP-W form a dimer of histone fold domains, and together CENP-TWSX forms a stable tetramer that can induce supercoils into DNA through a DNA-binding surface resembling that of a canonical nucleosome [115]. There are two types of CENP-T-based inner kinetochores: those which contain CENP-A (e.g. *G. gallus*) and those that lack CENP-A (e.g. *B. mori*), perhaps having replaced it with CENP-T.

#### 3.3.1. CENP-T-based kinetochores that contain CENP-A

Although *G. gallus* is not a traditional model organism, its kinetochore is relatively well studied, thanks in large part to work by the Fukagawa Lab. In chicken DT40 cells, both CENP-C and CENP-T can recruit the outer kinetochore to ectopic locations when artificially tethered to a LacO array [116]. But although the N-terminus of CENP-C is essential for CENP-C's interaction with Mis12c^MIND^ [116], it is not essential for viability or normal levels of Ndc80c recruitment to mitotic kinetochores [117], (just as the N-terminus of CENP-C^Mif2^ is not essential in budding yeast [94]). By contrast, the association of CENP-T with Ndc80c is essential for mitotic progression and recruitment of the outer kinetochore [117]. Using a talin sensor system, Hara *et al*. have even demonstrated that in native *G. gallus* kinetochores, pulling force from microtubules is exerted primarily on the CENP-T pathway [117]. In short, the CENP-C pathway is dispensable in *G. gallus*, while the CENP-T pathway is essential.

Intriguingly, forced binding of CENP-C to Mis12c^MIND^ (by removing the autoinhibitory basic motif of Dsn1) can rescue the growth defects observed in cells expressing a CENP-T mutant lacking its Ndc80c-binding domain [117]. This suggests that CENP-C might represent a supernumerary, backup Ndc80c recruitment pathway in the CENP-T-based inner kinetochore of *G. gallus*.

#### 3.3.2. CENP-T-based kinetochores that lack CENP-A

Until relatively recently, the centromere-specific histone variant CENP-A was thought to be the universal epigenetic determinant of centromere identity. Even this strategy is not, however, universal. CENP-A, although inferred to be present in the last eukaryotic common ancestor [30], has been independently lost in several lineages, including certain holocentric insects [27], kinetoplastids [118] and some fungi [29].

The Drinnenberg Lab has pioneered characterization of the CENP-A-independent *B. mori* kinetochore. Like *D. melanogaster*, *B. mori* has an inner kinetochore simplified by the loss of many CCAN components, but unlike fruit flies, whose spartan inner kinetochore is based on CENP-C, the silkworm appears to lack any CENP-C homologue [73]. Indeed, proteomic analysis in cell lines from holocentric Lepidoptera identified probable homologues only to CENP-I, -K, -L, -M, -N and -T in the inner kinetochore [119]. The identity of these potential kinetochore components was corroborated by the finding that their depletion caused severe mitotic defects. (Proteins with very remote homology to CENP-O, -P and -H were also identified, but not validated experimentally.) Curiously, the depletion of CENP-I, a protein that is not known to bind DNA in other organisms, caused the most severe phenotype, perhaps because it sits upstream of CENP-T in a tentative recruitment hierarchy [119]. Such a hierarchy would mirror the situation in budding yeast, where CENP-T^Cnn1^ is recruited to the kinetochore in a CENP-I^Ctf3^-dependent manner [113]. In *B. mori*, as in *G. gallus* cells [120], ectopic CENP-T tethered to a LacO array was sufficient to recruit outer kinetochore components, including Ndc80c [119].

The phenomenon of kinetochores lacking CENP-A is not confined to the order Lepidoptera, nor to holocentric organisms. Navarro-Mendoza and colleagues [29] have recently described the curious case of the early-diverging fungus *Mucor circinelloides*. This opportunistic human pathogen possesses clear homologues to nearly all the kinetochore components of *S. cerevisiae*, with the notable exceptions of CENP-A, -C and -QU [29]. Homologues of CENP-T and Mis12c^MIND^ components localize to kinetochores, but none of the histone H3 variants in *M. circinelloides* exhibit similar localization patterns, indicating that none of them has replaced CENP-A [29]. As CENP-A, -C and -QU appear to be missing from the entire order Mucorales, we speculate that this clade may represent an independently evolved, monocentric lineage in which CENP-T has functionally replaced CENP-A as the foundation of the kinetochore. Further biochemical characterization of Mucorales kinetochores will be needed to support this theory.

### 3.4. Hybrid inner kinetochores

Where do humans fall in this classification scheme? Genetic and biochemical evidence indicates that the human inner kinetochore is a ‘hybrid' of two of the types outlined above, dependent on *both* CENP-T and CENP-C (but not CENP-QU) for outer kinetochore recruitment.

Neither CENP-Q nor CENP-U is essential for viability in HeLa cells [121], although the latter *is* essential in embryonic cells [102]. These data, combined with the finding that human CENP-QU is incapable of binding Mis12c^MIND^ [101], strongly suggest that CENP-QU does not recruit the outer kinetochore in human cells.

By contrast, both the CENP-T and CENP-C genes are essential for growth and proliferation of human cancer cell lines [122], and there is excellent biochemical evidence for the dual roles of CENP-C and CENP-T. Both can recruit outer kinetochore proteins in human cells [123,124], and co-depletion of CENP-C and CENP-T in HeLa cells causes mislocalization of all other kinetochore components tested [120]. Conversely, simultaneous—but not individual—targeting of CENP-C and CENP-T to ectopic foci is sufficient to recruit outer kinetochore components at levels stoichiometrically proportional to the amount of CENP-C and -T present at the foci relative to kinetochores [124]. These foci are then able to interact with microtubules and perturb the segregation of chromosomes that also contain an endogenous kinetochore [124]. In short, *both* CENP-C and CENP-T are required for outer kinetochore assembly in human cells.

Intriguingly, using super-resolution fluorescence microscopy, Suzuki et al. [125] found that the mean position of the N-terminus of CENP-C in HeLa cells was surprisingly distant from Mis12c^MIND^, leading the authors to conclude that only a small minority of CENP-C binds Mis12^MIND^. Depletion of CENP-C increases intrakinetochore stretch of CENP-T [125], suggesting that the latter protein might shoulder more responsibility for force transmission when the CENP-C pathway is compromised.

*Xenopus laevis* is a model organism that also seems to rely on both CENP-C and CENP-T for Ndc80c recruitment. Immunodepletion of CENP-C from egg extracts prevents kinetochore formation on sperm chromatin [126]. *X. laevis* CENP-C binds directly to CENP-A nucleosomes and mediates Mis12^MIND^ recruitment to kinetochore [126,127]. These centromeres lack a CENP-Q homologue [30], but the CENP-T pathway also clearly contributes to outer kinetochore recruitment in *X. laevis.* Although depletion of CENP-C from *Xenopus* cell-free extracts reduces the kinetochore localization of CENP-TW, both Mis12c^MIND^ and Ndc80c are still recruited; importantly, this is not the case when CENP-T and CENP-C are co-depleted [128]. Although both pathways contribute to kinetochore recruitment, it could be argued that the contribution of CENP-C to kinetochore localization of CENP-TW indicates the primary importance of the former pathway.

As novel kinetochores are characterized at the biochemical level, it seems likely that other ‘hybrid' inner kinetochores will be discovered.

## 4. Conclusion

Though only a few model organisms' kinetochores have been biochemically characterized, at least three distinct strategies for bridging the centromere and outer kinetochore have emerged. *S. cerevisiae* relies primarily on CENP-QU^Okp1/Ame1^; *D. melanogaster* relies primarily on CENP-C; some organisms, including *G. gallus*, rely primarily on CENP-T. Intriguingly, there are now examples of multiple lineages that lack a canonical centromeric histone variant in which CENP-T may have taken its place as the foundation of the kinetochore. Compared with the near ubiquity of Ndc80c as the major microtubule coupler in every kinetochore that has been characterized biochemically, the diversity of these stratagems underscores an intriguing contrast between the evolutionarily labile inner kinetochore and highly conserved outer kinetochore.

Why such a diversity of inner kinetochore architectures? We hypothesize that it may be a consequence of centromere drive. Perhaps diverse inner kinetochore architectures have arisen over evolutionary time because inner kinetochore proteins must coevolve with rapidly changing centromeric DNA in order to retain their essential functions. This could even be framed as a corollary of the centromere drive theory: inner kinetochores, which mediate interaction with rapidly evolving centromeric DNA, should vary more in their composition and organization than outer kinetochores, which mediate interaction with microtubules, highly conserved elements of the cytoskeleton.

As more kinetochores are characterized biochemically, we predict that a still broader diversity of inner kinetochore architectures will be revealed. We are especially curious about the kinetochores of kinetoplastids, which lack known homologues to any CCAN proteins, and may also represent an exception to the universality of outer kinetochore architecture [28,71,129].

We also look forward to the characterization of more inner kinetochores from holocentric organisms. Holocentricity has been proposed as an adaptive mechanism to overcome the fitness costs of meiotic drive. It will therefore be of great interest whether or not the breakneck speed of inner kinetochore diversification has slowed in such species after the transition to holocentricity.

## Endnote

1 The exceptions are certain insects [27], kinetoplastids [28] and early-diverging fungi [29,30].

## Data accessibility

This article has no additional data.

## References

[1] Flemming W 1882 *Zellsubstanz, Kern und Zelltheilung*. Leipzig, Germany: F. C. W. Vogel.

[2] Aldrup-Macdonald ME, Sullivan BA 2014 The past, present, and future of human centromere genomics. Genes (Basel) 5, 33–50. (10.3390/genes5010033) 24683489 PMC3966626

[3] Miga KH 2015 Completing the human genome: the progress and challenge of satellite DNA assembly. Chromosome Res. 23, 421–426. (10.1007/s10577-015-9488-2) 26363799

[4] Miga KH, Newton Y, Jain M, Altemose N, Willard HF, Kent WJ 2014 Centromere reference models for human chromosomes X and Y satellite arrays. Genome Res. 24, 697–707. (10.1101/gr.159624.113) 24501022 PMC3975068

[5] Rudd MK, Willard HF 2004 Analysis of the centromeric regions of the human genome assembly. Trends Genet. 20, 529–533. (10.1016/j.tig.2004.08.008) 15475110

[6] Mandrioli M, Manicardi GC 2012 Unlocking holocentric chromosomes: new perspectives from comparative and functional genomics? Curr. Genomics 13, 343–349. (10.2174/138920212801619250) 23372420 PMC3401891

[7] Marques A, Pedrosa-Harand A 2016 Holocentromere identity: from the typical mitotic linear structure to the great plasticity of meiotic holocentromeres. Chromosoma 125, 669–681. (10.1007/s00412-016-0612-7) 27530342

[8] Melters DP, Paliulis LV, Korf IF, Chan SW 2012 Holocentric chromosomes: convergent evolution, meiotic adaptations, and genomic analysis. Chromosome Res. 20, 579–593. (10.1007/s10577-012-9292-1) 22766638

[9] Fukagawa T, Earnshaw WC 2014 The centromere: chromatin foundation for the kinetochore machinery. Dev. Cell 30, 496–508. (10.1016/j.devcel.2014.08.016) 25203206 PMC4160344

[10] Clarke L, Carbon J 1980 Isolation of a yeast centromere and construction of functional small circular chromosomes. Nature 287, 504–509. (10.1038/287504a0) 6999364

[11] Clarke L, Carbon J 1985 The structure and function of yeast centromeres. Annu. Rev. Genet. 19, 29–55. (10.1146/annurev.ge.19.120185.000333) 3909945

[12] Meraldi P, McAinsh AD, Rheinbay E, Sorger PK 2006 Phylogenetic and structural analysis of centromeric DNA and kinetochore proteins. Genome Biol. 7, R23 (10.1186/gb-2006-7-3-r23) 16563186 PMC1557759

[13] Pluta AF, Mackay AM, Ainsztein AM, Goldberg IG, Earnshaw WC 1995 The centromere: hub of chromosomal activities. Science 270, 1591–1594. (10.1126/science.270.5242.1591) 7502067

[14] Wiens GR, Sorger PK 1998 Centromeric chromatin and epigenetic effects in kinetochore assembly. Cell 93, 313–316. (10.1016/s0092-8674(00)81157-5) 9590163

[15] Allshire RC, Karpen GH 2008 Epigenetic regulation of centromeric chromatin: old dogs, new tricks? Nat. Rev. Genet. 9, 923–937. (10.1038/nrg2466) 19002142 PMC2586333

[16] Murillo-Pineda M, Jansen LET 2020 Genetics, epigenetics and back again: lessons learned from neocentromeres. Exp. Cell Res. 389, 111909 (10.1016/j.yexcr.2020.111909) 32068000

[17] Itoh Y, Kampf K, Balakrishnan CN, Arnold AP 2011 Karyotypic polymorphism of the zebra finch Z chromosome. Chromosoma 120, 255–264. (10.1007/s00412-010-0308-3) 21369954 PMC3099001

[18] Musilova P, Kubickova S, Vahala J, Rubes J 2013 Subchromosomal karyotype evolution in Equidae. Chromosome Res. 21, 175–187. (10.1007/s10577-013-9346-z) 23532666

[19] Purgato S et al. 2015 Centromere sliding on a mammalian chromosome. Chromosoma 124, 277–287. (10.1007/s00412-014-0493-6) 25413176 PMC4446527

[20] Lawrimore J, Bloom K 2019 The regulation of chromosome segregation via centromere loops. Crit. Rev. Biochem. Mol. Biol. 54, 352–370. (10.1080/10409238.2019.1670130) 31573359 PMC6856439

[21] Aze A, Sannino V, Soffientini P, Bachi A, Costanzo V 2016 Centromeric DNA replication reconstitution reveals DNA loops and ATR checkpoint suppression. Nat. Cell Biol. 18, 684–691. (10.1038/ncb3344) 27111843 PMC4939857

[22] Lawrimore J, Aicher JK, Hahn P, Fulp A, Kompa B, Vicci L, Falvo M, Taylor RM, Bloom K 2016 ChromoShake: a chromosome dynamics simulator reveals that chromatin loops stiffen centromeric chromatin. Mol. Biol. Cell 27, 153–166. (10.1091/mbc.E15-08-0575) 26538024 PMC4694754

[23] Lawrimore J, Doshi A, Friedman B, Yeh E, Bloom K 2018 Geometric partitioning of cohesin and condensin is a consequence of chromatin loops. Mol. Biol Cell 29, 2737–2750. (10.1091/mbc.E18-02-0131) 30207827 PMC6249845

[24] Paldi F, Alver B, Robertson D, Schalbetter SA, Kerr A, Kelly DA, Baxter J, Neale MJ, Marston AL 2020 Convergent genes shape budding yeast pericentromeres. Nature 582, 119–123. (10.1038/s41586-020-2244-6) 32494069 PMC7279958

[25] Yeh E, Haase J, Paliulis LV, Joglekar A, Bond L, Bouck D, Salmon ED, Bloom KS 2008 Pericentric chromatin is organized into an intramolecular loop in mitosis. Curr. Biol. 18, 81–90. (10.1016/j.cub.2007.12.019) 18211850 PMC2238682

[26] Henikoff S, Furuyama T 2010 Epigenetic inheritance of centromeres. Cold Spring Harb. Symp. Quant. Biol. 75, 51–60. (10.1101/sqb.2010.75.001) 21047902

[27] Drinnenberg IA, deYoung D, Henikoff S, Malik HS 2014 Recurrent loss of CenH3 is associated with independent transitions to holocentricity in insects. Elife 3, e03676 (10.7554/eLife.03676) PMC4359364 25247700

[28] Akiyoshi B, Gull K 2014 Discovery of unconventional kinetochores in kinetoplastids. Cell 156, 1247–1258. (10.1016/j.cell.2014.01.049) 24582333 PMC3978658

[29] Navarro-Mendoza MI et al. 2019 Early diverging fungus *Mucor circinelloides* lacks centromeric histone CENP-A and displays a mosaic of point and regional centromeres. Curr. Biol. 29, 3791–3802.e3796. (10.1016/j.cub.2019.09.024) 31679929 PMC6925572

[30] van Hooff JJ, Tromer E, van Wijk LM, Snel B, Kops GJ 2017 Evolutionary dynamics of the kinetochore network in eukaryotes as revealed by comparative genomics. EMBO Rep. 18, 1559–1571. (10.15252/embr.201744102) 28642229 PMC5579357

[31] Earnshaw WC, Rothfield N 1985 Identification of a family of human centromere proteins using autoimmune sera from patients with scleroderma. Chromosoma 91, 313–321. (10.1007/bf00328227) 2579778

[32] Keith KC, Baker RE, Chen Y, Harris K, Stoler S, Fitzgerald-Hayes M 1999 Analysis of primary structural determinants that distinguish the centromere-specific function of histone variant Cse4p from histone H3. Mol. Cell Biol. 19, 6130–6139. (10.1128/mcb.19.9.6130) 10454560 PMC84538

[33] Barnhart MC, Kuich PH, Stellfox ME, Ward JA, Bassett EA, Black BE, Foltz DR 2011 HJURP is a CENP-A chromatin assembly factor sufficient to form a functional de novo kinetochore. J. Cell Biol. 194, 229–243. (10.1083/jcb.201012017) 21768289 PMC3144403

[34] Black BE, Jansen LE, Maddox PS, Foltz DR, Desai AB, Shah JV, Cleveland DW 2007 Centromere identity maintained by nucleosomes assembled with histone H3 containing the CENP-A targeting domain. Mol. Cell 25, 309–322. (10.1016/j.molcel.2006.12.018) 17244537

[35] French BT, Westhorpe FG, Limouse C, Straight AF 2017 Xenopus laevis M18BP1 directly binds existing CENP-A nucleosomes to promote centromeric chromatin assembly. Dev. Cell 42, 190–199.e110. (10.1016/j.devcel.2017.06.021) 28743005 PMC5544353

[36] Fujita Y, Hayashi T, Kiyomitsu T, Toyoda Y, Kokubu A, Obuse C, Yanagida M 2007 Priming of centromere for CENP-a recruitment by human hMis18alpha, hMis18beta, and M18BP1. Dev. Cell 12, 17–30. (10.1016/j.devcel.2006.11.002) 17199038

[37] Hori T, Shang WH, Hara M, Ariyoshi M, Arimura Y, Fujita R, Kurumizaka H, Fukagawa T 2017 Association of M18BP1/KNL2 with CENP-a nucleosome is essential for centromere formation in non-mammalian vertebrates. Dev. Cell 42, 181–189.e183. (10.1016/j.devcel.2017.06.019) 28743004

[38] Tachiwana H, Müller S, Blümer J, Klare K, Musacchio A, Almouzni G 2015 HJURP involvement in de novo CenH3(CENP-A) and CENP-C recruitment. Cell Rep. 11, 22–32. (10.1016/j.celrep.2015.03.013) 25843710

[39] Zasadzinska E et al. 2018 Inheritance of CENP-A Nucleosomes during DNA Replication Requires HJURP. Dev. Cell 47, 348–362.e347. (10.1016/j.devcel.2018.09.003) 30293838 PMC6219920

[40] Hori T, Kagawa N, Toyoda A, Fujiyama A, Misu S, Monma N, Makino F, Ikeo K, Fukagawa T 2017 Constitutive centromere-associated network controls centromere drift in vertebrate cells. J. Cell Biol. 216, 101–113. (10.1083/jcb.201605001) 27940888 PMC5223601

[41] Gonzalez M, He H, Dong Q, Sun S, Li F 2014 Ectopic centromere nucleation by CENP-a in fission yeast. Genetics 198, 1433–1446. (10.1534/genetics.114.171173) 25298518 PMC4256763

[42] Palladino J, Chavan A, Sposato A, Mason TD, Mellone BG 2020 Targeted de novo centromere formation in drosophila reveals plasticity and maintenance potential of CENP-A chromatin. Dev. Cell 52, 379–394.e377. (10.1016/j.devcel.2020.01.005) 32049040 PMC7292339

[43] Scott KC, Sullivan BA 2014 Neocentromeres: a place for everything and everything in its place. Trends Genet. 30, 66–74. (10.1016/j.tig.2013.11.003) 24342629 PMC3913482

[44] Warburton PE 2004 Chromosomal dynamics of human neocentromere formation. Chromosome Res. 12, 617–626. (10.1023/B:CHRO.0000036585.44138.4b) 15289667

[45] McNulty SM, Sullivan BA 2017 Centromere silencing mechanisms. Prog. Mol. Subcell Biol. 56, 233–255. (10.1007/978-3-319-58592-5_10) 28840240 PMC13470870

[46] Stimpson KM, Matheny JE, Sullivan BA 2012 Dicentric chromosomes: unique models to study centromere function and inactivation. Chromosome Res. 20, 595–605. (10.1007/s10577-012-9302-3) 22801777 PMC3557915

[47] Malik HS, Henikoff S 2009 Major evolutionary transitions in centromere complexity. Cell 138, 1067–1082. (10.1016/j.cell.2009.08.036) 19766562

[48] Melters DP et al. 2013 Comparative analysis of tandem repeats from hundreds of species reveals unique insights into centromere evolution. Genome Biol. 14, R10 (10.1186/gb-2013-14-1-r10) 23363705 PMC4053949

[49] Henikoff S, Ahmad K, Malik HS 2001 The centromere paradox: stable inheritance with rapidly evolving DNA. Science 293, 1098–1102. (10.1126/science.1062939) 11498581

[50] Kursel LE, Malik HS 2018 The cellular mechanisms and consequences of centromere drive. Curr. Opin. Cell Biol. 52, 58–65. (10.1016/j.ceb.2018.01.011) 29454259 PMC5988936

[51] Malik HS, Henikoff S 2001 Adaptive evolution of Cid, a centromere-specific histone in *Drosophila*. Genetics 157, 1293–1298. 11238413 10.1093/genetics/157.3.1293 PMC1461554

[52] Borek WE et al 2020 The proteomic landscape of centromeric chromatin reveals an essential role for the Ctf19^CCAN^ complex in meiotic kinetochore assembly. *bioRxiv*, 2020.2006.2023.167395 (10.1101/2020.06.23.167395)

[53] Marston AL, Tham WH, Shah H, Amon A 2004 A genome-wide screen identifies genes required for centromeric cohesion. Science 303, 1367–1370. (10.1126/science.1094220) 14752166

[54] Vincenten N et al. 2015 The kinetochore prevents centromere-proximal crossover recombination during meiosis. Elife 4, e10850 (10.7554/eLife.10850) 26653857 PMC4749563

[55] Freitag M 2016 The kinetochore interaction network (KIN) of ascomycetes. Mycologia 108, 485–505. (10.3852/15-182) 26908646 PMC4864127

[56] Baker RE, Rogers K 2006 Phylogenetic analysis of fungal centromere H3 proteins. Genetics 174, 1481–1492. (10.1534/genetics.106.062794) 17028330 PMC1667059

[57] Ravi M et al. 2010 The rapidly evolving centromere-specific histone has stringent functional requirements in *Arabidopsis thaliana*. Genetics 186, 461–471. (10.1534/genetics.110.120337) 20628040 PMC2954480

[58] D'Archivio S, Wickstead B 2017 Trypanosome outer kinetochore proteins suggest conservation of chromosome segregation machinery across eukaryotes. J. Cell Biol. 216, 379–391. (10.1083/jcb.201608043) 28034897 PMC5294786

[59] Helgeson LA, Zelter A, Riffle M, MacCoss MJ, Asbury CL, Davis TN 2018 Human Ska complex and Ndc80 complex interact to form a load-bearing assembly that strengthens kinetochore-microtubule attachments. Proc. Natl Acad. Sci. USA 115, 2740–2745. (10.1073/pnas.1718553115) 29487209 PMC5856539

[60] Kim J, Zelter A, Umbreit NT, Bollozos A, Riffle M, Johnson R, MacCoss MJ, Asbury CL, Davis TN 2017 The Ndc80 complex bridges two Dam1 complex rings. Elife 6, e21069 (10.7554/eLife.21069) 28191870 PMC5354518

[61] Lampert F, Hornung P, Westermann S 2010 The Dam1 complex confers microtubule plus end-tracking activity to the Ndc80 kinetochore complex. J. Cell Biol. 189, 641–649. (10.1083/jcb.200912021) 20479465 PMC2872915

[62] Lampert F, Mieck C, Alushin GM, Nogales E, Westermann S 2013 Molecular requirements for the formation of a kinetochore-microtubule interface by Dam1 and Ndc80 complexes. J. Cell Biol. 200, 21–30. (10.1083/jcb.201210091) 23277429 PMC3542791

[63] McCleland ML, Gardner RD, Kallio MJ, Daum JR, Gorbsky GJ, Burke DJ, Stukenberg PT 2003 The highly conserved Ndc80 complex is required for kinetochore assembly, chromosome congression, and spindle checkpoint activity. Genes Dev. 17, 101–114. (10.1101/gad.1040903) 12514103 PMC195965

[64] Tien JF, Umbreit NT, Gestaut DR, Franck AD, Cooper J, Wordeman L, Gonen T, Asbury CL, Davis TN 2010 Cooperation of the Dam1 and Ndc80 kinetochore complexes enhances microtubule coupling and is regulated by aurora B. J. Cell Biol. 189, 713–723. (10.1083/jcb.200910142) 20479468 PMC2872917

[65] van Hooff JJE, Snel B, Kops G 2017 Unique phylogenetic distributions of the Ska and Dam1 complexes support functional analogy and suggest multiple parallel displacements of Ska by Dam1. Genome Biol. Evol. 9, 1295–1303. (10.1093/gbe/evx088) 28472331 PMC5439489

[66] Caldas GV, DeLuca KF, DeLuca JG 2013 KNL1 facilitates phosphorylation of outer kinetochore proteins by promoting Aurora B kinase activity. J. Cell Biol. 203, 957–969. (10.1083/jcb.201306054) 24344188 PMC3871439

[67] Cheeseman IM, Anderson S, Jwa M, Green EM, Kang JS, Yates JR III, Chan CS, Drubin DG, Barnes G 2002 Phospho-regulation of kinetochore-microtubule attachments by the Aurora kinase Ipl1p. Cell, 111, 163–172. (10.1016/s0092-8674(02)00973-x) 12408861

[68] Cheeseman IM, Chappie JS, Wilson-Kubalek EM, Desai A 2006 The conserved KMN network constitutes the core microtubule-binding site of the kinetochore. Cell 127, 983–997. (10.1016/j.cell.2006.09.039) 17129783

[69] Sarangapani KK, Akiyoshi B, Duggan NM, Biggins S, Asbury CL 2013 Phosphoregulation promotes release of kinetochores from dynamic microtubules via multiple mechanisms. Proc. Natl Acad. Sci. USA 110, 7282–7287. (10.1073/pnas.1220700110) 23589891 PMC3645574

[70] Umbreit NT, Miller MP, Tien JF, Ortolá JC, Gui L, Lee KK, Biggins S, Asbury CL, Davis TN 2014 Kinetochores require oligomerization of Dam1 complex to maintain microtubule attachments against tension and promote biorientation. Nat. Commun. 5, 4951 (10.1038/ncomms5951) 25236177 PMC4197110

[71] Llauró A, Hayashi H, Bailey ME, Wilson A, Ludzia P, Asbury CL, Akiyoshi B 2018 The kinetoplastid kinetochore protein KKT4 is an unconventional microtubule tip-coupling protein. J. Cell Biol. 217, 3886–3900. (10.1083/jcb.201711181) 30209069 PMC6219724

[72] Barth TK, Schade GO, Schmidt A, Vetter I, Wirth M, Heun P, Thomae AW, Imhof A 2014 Identification of novel *Drosophila* centromere-associated proteins. Proteomics 14, 2167–2178. (10.1002/pmic.201400052) 24841622

[73] Drinnenberg IA, Henikoff S, Malik HS 2016 Evolutionary turnover of kinetochore proteins: a ship of Theseus? Trends Cell Biol. 26, 498–510. (10.1016/j.tcb.2016.01.005) 26877204 PMC4914419

[74] Heeger S, Leismann O, Schittenhelm R, Schraidt O, Heidmann S, Lehner CF 2005 Genetic interactions of separase regulatory subunits reveal the diverged *Drosophila* Cenp-C homolog. Genes Dev. 19, 2041–2053. (10.1101/gad.347805) 16140985 PMC1199574

[75] Przewloka MR, Zhang W, Costa P, Archambault V, D'Avino PP, Lilley KS, Laue ED, McAinsh AD, Glover DM 2007 Molecular analysis of core kinetochore composition and assembly in *Drosophila melanogaster*. PLoS ONE 2, e478 (10.1371/journal.pone.0000478) 17534428 PMC1868777

[76] Mellone BG, Grive KJ, Shteyn V, Bowers SR, Oderberg I, Karpen GH 2011 Assembly of *Drosophila* centromeric chromatin proteins during mitosis. PLoS Genet 7, e1002068 (10.1371/journal.pgen.1002068) 21589899 PMC3093364

[77] Przewloka MR, Venkei Z, Bolanos-Garcia VM, Debski J, Dadlez M, Glover DM 2011 CENP-C is a structural platform for kinetochore assembly. Curr. Biol. 21, 399–405. (10.1016/j.cub.2011.02.005) 21353555

[78] Richter MM, Poznanski J, Zdziarska A, Czarnocki-Cieciura M, Lipinszki Z, Dadlez M, Glover DM, Przewloka MR 2016 Network of protein interactions within the *Drosophila* inner kinetochore. Open Biol. 6, 150238 (10.1098/rsob.150238) 26911623 PMC4772809

[79] Ye AA, Cane S, Maresca TJ 2016 Chromosome biorientation produces hundreds of piconewtons at a metazoan kinetochore. Nat. Commun. 7, 13221 (10.1038/ncomms13221) 27762268 PMC5080440

[80] Singh TR et al. 2010 MHF1-MHF2, a histone-fold-containing protein complex, participates in the Fanconi anemia pathway via FANCM. Mol. Cell 37, 879–886. (10.1016/j.molcel.2010.01.036) 20347429 PMC2848122

[81] Yan Z et al. 2010 A histone-fold complex and FANCM form a conserved DNA-remodeling complex to maintain genome stability. Mol. Cell 37, 865–878. (10.1016/j.molcel.2010.01.039) 20347428 PMC2847587

[82] Desai A, Rybina S, Muller-Reichert T, Shevchenko A, Hyman A, Oegema K 2003 KNL-1 directs assembly of the microtubule-binding interface of the kinetochore in *C. elegans*. Genes Dev. 17, 2421–2435. (10.1101/gad.1126303) 14522947 PMC218079

[83] Oegema K, Desai A, Rybina S, Kirkham M, Hyman AA 2001 Functional analysis of kinetochore assembly in *Caenorhabditis elegans*. J. Cell Biol. 153, 1209–1226. (10.1083/jcb.153.6.1209) 11402065 PMC2192036

[84] Keyes BE, Sykes KD, Remington CE, Burke DJ 2012 Sister chromatids segregate at mitosis without mother–daughter bias in *Saccharomyces cerevisiae*. Genetics 192, 1553–1557. (10.1534/genetics.112.145680) 23051643 PMC3512160

[85] Thorpe PH, Bruno J, Rothstein R 2009 Kinetochore asymmetry defines a single yeast lineage. Proc. Natl Acad. Sci. USA 106, 6673–6678. (10.1073/pnas.0811248106) 19346480 PMC2672522

[86] Bensasson D, Zarowiecki M, Burt A, Koufopanou V 2008 Rapid evolution of yeast centromeres in the absence of drive. Genetics 178, 2161–2167. (10.1534/genetics.107.083980) 18430941 PMC2323805

[87] Kobayashi N, Suzuki Y, Schoenfeld LW, Müller CA, Nieduszynski C, Wolfe KH, Tanaka TU 2015 Discovery of an unconventional centromere in budding yeast redefines evolution of point centromeres. Curr. Biol. 25, 2026–2033. (10.1016/j.cub.2015.06.023) 26166782 PMC4533239

[88] Padmanabhan S, Thakur J, Siddharthan R, Sanyal K 2008 Rapid evolution of Cse4p-rich centromeric DNA sequences in closely related pathogenic yeasts, *Candida albicans* and *Candida dubliniensis*. Proc. Natl Acad. Sci. USA 105, 19 797–19 802. (10.1073/pnas.0809770105) PMC2604992 19060206

[89] Biggins S 2013 The composition, functions, and regulation of the budding yeast kinetochore. Genetics 194, 817–846. (10.1534/genetics.112.145276) 23908374 PMC3730914

[90] Ortiz J, Stemmann O, Rank S, Lechner J 1999 A putative protein complex consisting of Ctf19, Mcm21, and Okp1 represents a missing link in the budding yeast kinetochore. Genes Dev. 13, 1140–1155. (10.1101/gad.13.9.1140) 10323865 PMC316948

[91] Meluh PB, Koshland D 1995 Evidence that the MIF2 gene of *Saccharomyces cerevisiae* encodes a centromere protein with homology to the mammalian centromere protein CENP-C. Mol. Biol. Cell 6, 793–807. (10.1091/mbc.6.7.793) 7579695 PMC301241

[92] Westermann S, Drubin DG, Barnes G 2007 Structures and functions of yeast kinetochore complexes. Annu. Rev. Biochem. 76, 563–591. (10.1146/annurev.biochem.76.052705.160607) 17362199

[93] Westermann S, Schleiffer A 2013 Family matters: structural and functional conservation of centromere-associated proteins from yeast to humans. Trends Cell Biol. 23, 260–269. (10.1016/j.tcb.2013.01.010) 23481674

[94] Hornung P et al. 2014 A cooperative mechanism drives budding yeast kinetochore assembly downstream of CENP-A. J. Cell Biol. 206, 509–524. (10.1083/jcb.201403081) 25135934 PMC4137059

[95] Killinger K et al 2020 Auto-inhibition of Mif2/CENP-C ensures centromere-dependent kinetochore assembly in budding yeast. Embo J. 39, e102938 (10.15252/embj.2019102938) 32515113 PMC7360964

[96] Hamilton GE, Helgeson LA, Noland CL, Asbury CL, Dimitrova YN, Davis TN 2020 Reconstitution reveals two paths of force transmission through the kinetochore. Elife 9, e56582 (10.7554/eLife.56582) 32406818 PMC7367685

[97] Anedchenko EA et al. 2019 The kinetochore module Okp1 CENP-Q /Ame1 CENP-U is a reader for N-terminal modifications on the centromeric histone Cse4 CENP-A. EMBO J. 38, e98991 (10.15252/embj.201898991) 30389668 PMC6315295

[98] Hayles J, Wood V, Jeffery L, Hoe KL, Kim DU, Park HO, Salas-Pino S, Heichinger C, Nurse P 2013 A genome-wide resource of cell cycle and cell shape genes of fission yeast. Open Biol. 3, 130053 (10.1098/rsob.130053) 23697806 PMC3866870

[99] Kim DU et al. 2010 Analysis of a genome-wide set of gene deletions in the fission yeast *Schizosaccharomyces pombe*. Nat. Biotechnol. 28, 617–623. (10.1038/nbt.1628) 20473289 PMC3962850

[100] Amaro AC, Samora CP, Holtackers R, Wang E, Kingston IJ, Alonso M, Lampson M, McAinsh AD, Meraldi P 2010 Molecular control of kinetochore–microtubule dynamics and chromosome oscillations. Nat. Cell Biol. 12, 319–329. (10.1038/ncb2033) 20228811 PMC2909587

[101] Pesenti ME et al. 2018 Reconstitution of a 26-subunit human kinetochore reveals cooperative microtubule binding by CENP-OPQUR and NDC80. Mol. Cell 71, 923–939. (10.1016/j.molcel.2018.07.038) 30174292 PMC6162344

[102] Kagawa N, Hori T, Hoki Y, Hosoya O, Tsutsui K, Saga Y, Sado T, Fukagawa T 2014 The CENP-O complex requirement varies among different cell types. Chromosome Res. 22, 293–303. (10.1007/s10577-014-9404-1) 24481920 PMC4129241

[103] Kang YH, Park CH, Kim TS, Soung NK, Bang JK, Kim BY, Park JE, Lee KS 2011 Mammalian polo-like kinase 1-dependent regulation of the PBIP1-CENP-Q complex at kinetochores. J. Biol. Chem. 286, 19 744–19 757. (10.1074/jbc.M111.224105) PMC3103353 21454580

[104] Kang YH et al. 2006 Self-regulated Plk1 recruitment to kinetochores by the Plk1-PBIP1 interaction is critical for proper chromosome segregation. Mol. Cell 24, 409–422. (10.1016/j.molcel.2006.10.016) 17081991

[105] Bancroft J, Auckland P, Samora CP, McAinsh AD 2015 Chromosome congression is promoted by CENP-Q- and CENP-E-dependent pathways. J. Cell Sci. 128, 171–184. (10.1242/jcs.163659) 25395579 PMC4282051

[106] Tromer EC, van Hooff JJE, Kops G, Snel B 2019 Mosaic origin of the eukaryotic kinetochore. Proc. Natl Acad. Sci. USA 116, 12 873–12 882. (10.1073/pnas.1821945116) PMC6601020 31127038

[107] Bock LJ et al. 2012 Cnn1 inhibits the interactions between the KMN complexes of the yeast kinetochore. Nat. Cell Biol. 14, 614–624. (10.1038/ncb2495) 22561345 PMC3438452

[108] De Wulf P, McAinsh AD, Sorger PK 2003 Hierarchical assembly of the budding yeast kinetochore from multiple subcomplexes. Genes Dev. 17, 2902–2921. (10.1101/gad.1144403) 14633972 PMC289150

[109] Giaever G et al. 2002 Functional profiling of the *Saccharomyces cerevisiae* genome. Nature 418, 387–391. (10.1038/nature00935) 12140549

[110] Schleiffer A, Maier M, Litos G, Lampert F, Hornung P, Mechtler K, Westermann S 2012 CENP-T proteins are conserved centromere receptors of the Ndc80 complex. Nat. Cell Biol. 14, 604–613. (10.1038/ncb2493) 22561346

[111] Yuen KW, Warren CD, Chen O, Kwok T, Hieter P, Spencer FA 2007 Systematic genome instability screens in yeast and their potential relevance to cancer. Proc. Natl Acad. Sci. USA 104, 3925–3930. (10.1073/pnas.0610642104) 17360454 PMC1820685

[112] Lang J, Barber A, Biggins S 2018 An assay for de novo kinetochore assembly reveals a key role for the CENP-T pathway in budding yeast. Elife 7, e37819 (10.7554/eLife.37819) 30117803 PMC6097842

[113] Pekgöz ltunkaya G, Malvezzi F, Demianova Z, Zimniak T, Litos G, Weissmann F, Mechtler K, Herzog F, Westermann S 2016 CCAN assembly configures composite binding interfaces to promote cross-linking of Ndc80 complexes at the kinetochore. Curr. Biol. 26, 2370–2378. (10.1016/j.cub.2016.07.005) 27524485

[114] Hori T et al. 2008 CCAN makes multiple contacts with centromeric DNA to provide distinct pathways to the outer kinetochore. Cell 135, 1039–1052. (10.1016/j.cell.2008.10.019) 19070575

[115] Nishino T, Takeuchi K, Gascoigne KE, Suzuki A, Hori T, Oyama T, Morikawa K, Cheeseman IM, Fukagawa T 2012 CENP-T-W-S-X forms a unique centromeric chromatin structure with a histone-like fold. Cell 148, 487–501. (10.1016/j.cell.2011.11.061) 22304917 PMC3277711

[116] Hori T, Shang WH, Takeuchi K, Fukagawa T 2013 The CCAN recruits CENP-A to the centromere and forms the structural core for kinetochore assembly. J. Cell Biol. 200, 45–60. (10.1083/jcb.201210106) 23277427 PMC3542802

[117] Hara M, Ariyoshi M, Okumura EI, Hori T, Fukagawa T 2018 Multiple phosphorylations control recruitment of the KMN network onto kinetochores. Nat. Cell Biol. 20, 1378–1388. (10.1038/s41556-018-0230-0) 30420662

[118] Lowell JE, Cross GA 2004 A variant histone H3 is enriched at telomeres in *Trypanosoma brucei*. J. Cell Sci. 117(Pt 24), 5937–5947. (10.1242/jcs.01515) 15522895

[119] Cortes-Silva N et al. 2020 CenH3-Independent kinetochore assembly in Lepidoptera requires CCAN, Including CENP-T. Curr. Biol. 30, 561–572.e510. (10.1016/j.cub.2019.12.014) 32032508

[120] Gascoigne KE, Takeuchi K, Suzuki A, Hori T, Fukagawa T, Cheeseman IM 2011 Induced ectopic kinetochore assembly bypasses the requirement for CENP-A nucleosomes. Cell 145, 410–422. (10.1016/j.cell.2011.03.031) 21529714 PMC3085131

[121] Okada M, Cheeseman IM, Hori T, Okawa K, McLeod IX, Yates JR, Desai A, Fukagawa T 2006 The CENP-H-I complex is required for the efficient incorporation of newly synthesized CENP-A into centromeres. Nat. Cell Biol. 8, 446–457. (10.1038/ncb1396) 16622420

[122] Wang T, Birsoy K, Hughes NW, Krupczak KM, Post Y, Wei JJ, Lander ES, Sabatini DM 2015 Identification and characterization of essential genes in the human genome. Science 350, 1096–1101. (10.1126/science.aac7041) 26472758 PMC4662922

[123] Petrovic A et al. 2016 Structure of the MIS12 complex and molecular basis of its interaction with CENP-C at human kinetochores. Cell 167, 1028–1040. (10.1016/j.cell.2016.10.005) 27881301 PMC5101189

[124] Rago F, Gascoigne KE, Cheeseman IM 2015 Distinct organization and regulation of the outer kinetochore KMN network downstream of CENP-C and CENP-T. Curr. Biol. 25, 671–677. (10.1016/j.cub.2015.01.059) 25660545 PMC4348146

[125] Suzuki A, Badger BL, Wan X, DeLuca JG, Salmon ED 2014 The architecture of CCAN proteins creates a structural integrity to resist spindle forces and achieve proper Intrakinetochore stretch. Dev. Cell 30, 717–730. (10.1016/j.devcel.2014.08.003) 25268173 PMC4237209

[126] Milks KJ, Moree B, Straight AF 2009 Dissection of CENP-C-directed centromere and kinetochore assembly. Mol. Biol. Cell 20, 4246–4255. (10.1091/mbc.e09-05-0378) 19641019 PMC2754938

[127] Carroll CW, Milks KJ, Straight AF 2010 Dual recognition of CENP-A nucleosomes is required for centromere assembly. J. Cell Biol. 189, 1143–1155. (10.1083/jcb.201001013) 20566683 PMC2894454

[128] Krizaic I, Williams SJ, Sánchez P, Rodríguez-Corsino M, Stukenberg PT, Losada A 2015 The distinct functions of CENP-C and CENP-T/W in centromere propagation and function in *Xenopus* egg extracts. Nucleus 6, 133–143. (10.1080/19491034.2014.1003509) 25569378 PMC4615894

[129] Akiyoshi B 2016 The unconventional kinetoplastid kinetochore: from discovery toward functional understanding. Biochem. Soc. Trans. 44, 1201–1217. (10.1042/bst20160112) 27911702 PMC5095916

